# MicroRNAs Targeting HIF-2α, VEGFR1 and/or VEGFR2 as Potential Predictive Biomarkers for VEGFR Tyrosine Kinase and HIF-2α Inhibitors in Metastatic Clear-Cell Renal Cell Carcinoma

**DOI:** 10.3390/cancers13123099

**Published:** 2021-06-21

**Authors:** Lisa Kinget, Eduard Roussel, Annelies Verbiest, Maarten Albersen, Cristina Rodríguez-Antona, Osvaldo Graña-Castro, Lucía Inglada-Pérez, Jessica Zucman-Rossi, Gabrielle Couchy, Sylvie Job, Aurélien de Reyniès, Annouschka Laenen, Marcella Baldewijns, Benoit Beuselinck

**Affiliations:** 1 Department of General Medical Oncology, Leuven Cancer Institute, University Hospitals Leuven, 3000 Leuven, Belgium; lisa.kinget@uzleuven.be (L.K.); annelies.verbiest@uzleuven.be (A.V.); 2 Department of Urology, University Hospitals Leuven, 3000 Leuven, Belgium; eduard.roussel@uzleuven.be (E.R.); maarten.albersen@uzleuven.be (M.A.); 3 Centro de Investigación Biomédica en Red de Enfermedades Raras (CIBERER), 28029 Madrid, Spain; crodriguez@cnio.es (C.R.-A.); ograna@cnio.es (O.G.-C.); 4 Department of Statistics and Operational Research, Faculty of Medicine, Complutense University, 28040 Madrid, Spain; lucia.inglada.perez@ucm.es; 5 Centre de Recherche des Cordeliers, Sorbonne Université, Université de Paris, INSERM, Functional Genomics of Solid Tumors Laboratory, équipe Labellisée Ligue Nationale Contre le Cancer, Labex OncoImmunology, F-75006 Paris, France; jessica.zucman-rossi@inserm.fr (J.Z.-R.); gabrielle.couchy@inserm.fr (G.C.); 6 Programme Cartes d’Identité des Tumeurs, Ligue Nationale Contre le Cancer, F-75006 Paris, France; sylvie.job@ligue-cancer.net (S.J.); aurelien.dereynies@ligue-cancer.net (A.d.R.); 7 Department of Biostatistics, KU Leuven, 3000 Leuven, Belgium; annouschka.laenen@kuleuven.be; 8 Department of Pathology, University Hospitals Leuven, 3000 Leuven, Belgium; marcella.baldewijns@uzleuven.be

**Keywords:** microRNAs, HIF-2α, VEGFR2, renal cell carcinoma, miR-34c-5p, miR-185-5p, miR-221-3p, miR-222-3p, miR-223-3p, miR-3529-3p

## Abstract

**Simple Summary:**

Metastatic clear-cell renal cell carcinoma is characterized by heightened angiogenesis through increased expression of HIF-2α and VEGFR2. VEGFR tyrosine kinase inhibitors are a cornerstone of metastatic clear-cell renal cell carcinoma treatment, and new treatments targeting HIF-2α are currently under investigation. However, clinically useful biomarkers that can predict response to these treatments are lacking. MicroRNAs are small RNA molecules that interfere with gene translation. In this study, we identified four microRNAs that potentially interfere with the translation of VEGFR1 and/or VEGFR2 and are associated with tumor shrinkage and progression-free survival upon treatment with VEGFR-TKIs. These microRNAs might be predictive of response to VEGFR-TKIs. Moreover, we identified three microRNAs associated with HIF-2α expression and with tumor shrinkage and progression-free survival upon treatment with VEGFR-TKIs. These three microRNAs might be able to predict response not only to treatment with VEGFR-TKIs but possibly also to treatment with the upcoming HIF-2α inhibitor belzutifan.

**Abstract:**

Metastatic clear-cell renal cell carcinoma (m-ccRCC) is characterized by increased hypoxia-induced factor (HIF)-2α and vascular endothelial growth factor receptor (VEGFR)-dependent angiogenesis through loss of function of the von Hippel–Lindau protein. VEGFR tyrosine kinase inhibitors (VEGFR-TKIs) are a cornerstone of m-ccRCC treatment, and new treatments targeting HIF-2α are currently under investigation. However, predictive biomarkers for these treatments are lacking. In this retrospective cohort study including 109 patients treated with VEGFR-targeted therapies as first-line treatment, we aimed to study the possible predictive function of microRNAs (miRNAs) targeting HIF-2α, VEGFR1 and VEGFR2. We selected miRNAs inversely correlated with HIF-2α, VEGFR1 and/or VEGFR2 expression and with predicted target sites in the respective genes and subsequently studied their impact on therapeutic outcomes. We identified four miRNAs (miR-34c-5p, miR-221-3p, miR-222-3p and miR-3529-3p) inversely correlated with VEGFR1 and/or VEGFR2 expression and associated with tumor shrinkage and progression-free survival (PFS) upon treatment with VEGFR-TKIs, highlighting the potential predictive value of these miRNAs. Moreover, we identified three miRNAs (miR-185-5p, miR-223-3p and miR-3529-3p) inversely correlated with HIF-2α expression and associated with tumor shrinkage and PFS upon treatment with VEGFR-TKIs. These three miRNAs can have a predictive value not only upon treatment with VEGFR-TKIs but possibly also upon treatment with the upcoming HIF-2α inhibitor belzutifan.

## 1. Introduction

Renal cell carcinoma (RCC) accounts for 3% of all malignancies worldwide. Clear-cell RCC (ccRCC) is the most frequent subtype and also holds the highest cancer-related mortality [1]. One of the main oncogenic drivers of ccRCC is loss of function of the von Hippel–Lindau (VHL) gene, which results in a continuous downstream activation of hypoxia-induced factors (HIFs) such as HIF-2α and subsequent overexpression of hypoxia-induced genes such as the pro-angiogenic vascular endothelial growth factor (VEGF) [2]. This activation of the HIF-VEGF pathway leads to characteristic hypervascularization of ccRCC tumors and is intensively studied as a drug target for this chemotherapy-resistant malignancy. Angiogenesis inhibitors such as VEGF receptor tyrosine kinase inhibitors (VEGFR-TKIs) revolutionized the treatment of metastatic ccRCC (m-ccRCC) in 2006 [3] and remain an essential component of current management strategies, both in combination with immuno-oncology first-line therapies or as single agents in further treatment lines. Although HIF-2α was long considered a non-druggable target like other transcription factors, recent advances have led to the development of small-molecule inhibitors preventing its dimerization and effectively preventing transcription of HIF-2α-responsive genes [4]. Of these, PT-2385 and later MK-6482 (belzutifan) are currently being investigated in clinical trials [5,6,7]. Despite these therapeutic advances, a strong clinical need remains for biomarkers that allow selection of patients who might benefit from these agents [8].

microRNAs (miRNAs) are small non-protein coding RNA molecules that have been intensely investigated for personalized medicine strategies. They are important drivers of posttranscriptional gene regulation and suppress protein translation by acceleration of mRNA degradation or interference with translation. Dysregulation of their expression is frequently implicated in oncogenesis. The ability to quantify miRNAs not only in tumoral tissues but also in urinary and serum samples further highlights their biomarker potential. In ccRCC, several miRNAs are differentially expressed between normal tissues or benign diseases and malignant tumors, and they can therefore aid in diagnosis [9,10]. The use of miRNAs as prognostic markers is extensively studied [11,12,13]. Several studies have also investigated the predictive value of miRNAs in m-ccRCC. Go et al. proposed a response classifier for VEGFR-TKIs that included five miRNAs [14]. The miR-221/222 cluster predicts response to sunitinib treatment [15]. Gámez-Pozo et al. described miRNA signatures associated with resistance to first-line treatment with sunitinib [16].

In this study, we aimed to investigate miRNAs targeting HIF-2α, VEGFR1 and VEGFR2 in m-ccRCC as potential predictive biomarkers for treatment efficacy of anti-angiogenic therapies in m-ccRCC. As HIF-2α, VEGFR1 and VEGFR2 are the main drug targets of anti-angiogenic therapies in m-ccRCC, high expression of miRNAs that target them would lead to decreased translation of HIF-2α, VEGFR1 or VEGFR2, possibly resulting in decreased therapeutic efficiency of HIF-2α inhibitors or VEGFR-TKIs.

## 2. Materials and Methods

### 2.1. Patient Selection

A retrospective study including m-ccRCC patients who received VEGFR-TKIs as first-line treatment was performed in the University Hospitals of Leuven. First therapeutic intervention was nephrectomy for all patients. Ethical approval was granted by the Ethics Committee Research of UZ/KULeuven (approval number S53479/S63833). The institutional board granted approval for use of tissue samples of deceased patients. All patients provided written informed consent.

### 2.2. Study Objectives and Endpoints

The principal objective was to identify miRNAs targeting HIF-2α, VEGFR1 and VEGFR2 mRNA with a possible predictive value upon treatment with VEGFR-TKIs. The secondary objective was to propose miRNAs targeting HIF-2α as potential future predictive biomarkers upon treatment with the HIF-2α inhibitor belzutifan. Primary endpoints were tumor shrinkage upon treatment with VEGFR-TKIs as measured by Response Evaluation Criteria In Solid Tumors, version 1.1 (RECIST v.1.1) and progression-free survival (PFS) from start of VEGFR-TKIs. Tumor shrinkage is a primary endpoint in this study as it is rarely the spontaneous evolution of a tumor and more likely the result of therapeutic efficacy. Association with tumor shrinkage is therefore indicative of a potential predictive impact of a biomarker, whereas association with increased PFS upon treatment with VEGFR-TKIs can be a consequence of either indolent disease or treatment efficacy. The secondary endpoint was overall survival (OS) since start of first-line therapy. Additionally, we aimed to investigate whether expression of these miRNAs differs between previously described molecular subtypes in ccRCC, as their prognostic value and their predictive value upon treatment with VEGFR-TKIs has been well described [17,18].

### 2.3. miRNA Extraction and Next-Generation Sequencing (NGS)

As described previously, miRNA was extracted from FFPE tumoral slides at the Centro Nacional de Investigaciones Oncológicas (Madrid) [19]. After pathological review of H&E-stained slides for confirmation of diagnosis and estimation of tumoral content, blank tumor slides were processed for total RNA extraction with the Recover All Total Nucleic Acid Isolation kit (Ambion, Thermo Fisher Scientific, Waltham, MA, USA). After assessment of RNA concentration and quality using a NanoDrop Spectrophotometer (Nanodrop Technologies, Wilmington, DE, USA), cDNA libraries were synthesized from 500 ng of total RNA using the NEBNext Multiplex Small RNA Library Prep for Illumina (New England Biolabs, Ipswich, MA, USA) as per the manufacturer’s protocol. The libraries were next sequenced for 50 bases in a single-read format (Genome Analyzer IIX, Illumina, San Diego, CA, USA). Quality control of the reads was performed using FastQC software (https://www.bioinformatics.babraham.ac.uk/projects/fastqc/, accessed on 19 May 2021). Reads with lengths between 15 and 35 bp were withheld. Cutadapt v1.2.1 (http://journal.embnet.org/index.php/embnetjournal/article/view/200, accessed on 19 May 2021) was used for adapter sequence removal. Genome alignment was performed using Bowtie 0.12.7 (Johns Hopkins University, Baltimore, MD, USA) and Samtools 0.1.18 (http://samtools.sourceforge.net, accessed on 19 May 2021). Subsequently, HTSeq v0.5.3p9 (https://pypi.org/project/HTSeq/, accessed on 19 May 2021) using the miRbase v20 annotation for hg19 was used for determining raw counts. For a total of 2589 miRNAs, raw counts were obtained, which were then normalized using the DESeq Bioconductor package in R (v3.1.2) (R Core Team, Vienna, Austria). miRNA with low expression levels (0 counts per million reads in over 100 samples) were omitted, resulting in expression values for 454 miRNAs.

### 2.4. mRNA Extraction and qRT-PCR

RNA extraction was performed for patients for whom fresh frozen tumoral tissues were available, as previously described [18,20]. RNA was quantified with the NanoDrop spectrophotometer (Thermo Fisher Scientific, Waltham, MA, USA), and quality control was performed using gel electrophoresis (E-gel 48 1% agarose and Mother E-base by Invitrogen, Waltham, MA, USA). Using a High Capacity cDNA Reverse Transcription Kit (Life Technologies, Carlsbad, CA, USA), 0.5 µg of total RNA was reverse transcribed into a final volume of 50 µL. Quantitative reverse transcription-polymerase chain reaction (qRT-PCR) was performed according to the manufacturer’s instructions using the BioMark qRT-PCR system (Fluidigm, South San Francisco, CA, USA). A total of 6 ng of cDNA per sample was preamplified by using the PreAmp Master Mix (Thermo Fisher Scientific, Waltham, MA, USA). After preparation of a primer mix as reported in our previous study [20], 1 µL of the primer mixture was combined with 1 µL of TaqMan PreAmp Master Mix (Thermo Fisher Scientific, Waltham, MA, USA) and 3 µL of diluted cDNA (2 ng/µL). This mixture was subsequently incubated at 95 °C for 2 min and then 14 cycles of 95 °C for 15 sec and 60 °C for 4 min. Following a 1:5 dilution of preamplified cDNA in DNase/RNase free water, qRT-PCR was run in the Dynamic Array Integrated Fluidic Circuits on the Biomark HD system (Fluidigm, South San Francisco, CA, USA). Subsequently, 3.5 µL of Taqman Gene Expression Master Mix (Applied Biosystems, Foster City, CA, USA) was mixed with 0.35 µL 20X GE Sample Loading Reagent (Fluidigm, South San Francisco, CA, USA). After adding 3.15 µL of diluted PreAmplified cDNA, 5µL of this combination was loaded into the wells of 96.96 Dynamic Arrays. A BioMark HD reader (Fluidigm, South San Francisco, CA, USA) was next used to perform qRT-PCR. Using the Fluidigm qRT-PCR Analysis software (4.1.3) (Fluidigm, South San Francisco, CA, USA), mRNA expression levels (threshold cycle values) were calculated. Beta-actin expression levels were used for data normalization.

### 2.5. Selection of miRNAs

To identify miRNAs potentially targeting HIF-2α, VEGFR1 and VEGFR2, we performed a two-step selection: first, we performed a correlation analysis between mRNA expression of HIF-2α, VEGFR1 and VEGFR2 and all miRNAs (*n* = 454), using Spearman correlation. For this, we used the molecular data of patients for whom both miRNA and mRNA data were available (*n* = 76). Correction for multiple testing was performed using the Benjamini–Hochberg method [21]. As miRNA expression values were obtained through NGS, higher values correspond with higher expression. mRNA expression is reported as Ct values, and higher values correspond with lower expression. We therefore selected correlations with a positive correlation coefficient, which additionally had a false discovery rate (FDR) adjusted *p*-value < 0.05. Next, we searched three known miRNA–mRNA interaction databases for miRNAs potentially targeting HIF-2α, VEGFR1 and/or VEGFR2 (miRDB (http://mirdb.org/, accessed on 19 May 2021) and miRabel (http://bioinfo.univ-rouen.fr/mirabel/index.php?page=mir, accessed on 19 May 2021), databases for prediction of functional miRNA targets [22,23], and DIANA TarBase v8 (https://carolina.imis.athena-innovation.gr/diana_tools/web/index.php?r=tarbasev8%2Findex, accessed on 19 May 2021), a database of experimentally validated miRNA-target pairs [24]). For miRabel, the cutoff of 0.05 for the predicted RRA score was used, as recommended by the authors [23]. miRNAs that were both correlated to one of the mRNAs and for which this interaction was listed in one of previously mentioned databases were withheld for further analysis. Using the miRNAs we selected during these steps, we analyzed the impact of miRNA expression on PFS and OS using Cox proportional hazards regression models. Descriptive statistics for time-to-event variables are based on Kaplan–Meier estimates. Tumor shrinkage was measured using RECIST v1.1. For each patient, we noted the maximal tumor shrinkage upon treatment compared to baseline. In case of early progressive disease with progression outside target lesions, we assigned 21% of progression compared to baseline. Analyses were performed using R (version 4.0.03) (R Core Team, Vienna, Austria) and SAS software (version 9.4) (SAS Institute Inc., Cary, NC, USA).

### 2.6. Molecular Subtypes

We previously described a classification using unsupervised clustering to classify m-ccRCC tumors [18]. For patients with mRNA expression data available, the molecular subtypes were previously determined and were used for further analysis.

### 2.7. Clinical Data

Staging with computed tomography of the chest and abdomen was performed every 2 to 3 months during treatment with VEGFR-TKIs. Pathology slides underwent expert genitourinary pathology review. The International Metastatic RCC Database Consortium (IMDC) prognostic category was retrospectively assessed [25].

## 3. Results

### 3.1. Included Patients

In total, 109 patients treated with first-line VEGFR-TKIs and with available miRNA sequencing data were included (Table S1). Patient characteristics are described in Table 1. The majority of patients were treated with first-line sunitinib (62.4%); the remaining patients were treated with sorafenib (27.5%) or pazopanib (10.1%). For 76 of the 109 patients, mRNA expression data from fresh frozen samples were available.

### 3.2. miRNAs Potentially Targeting HIF-2α, VEGFR1 and/or VEGFR2

**Figure 1:**
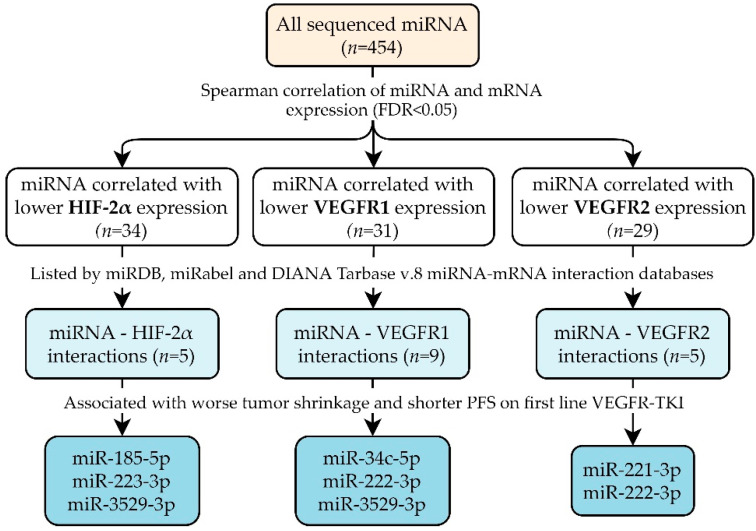
Through correlation analysis and miRNA–mRNA interaction databases, 14 miRNAs were identified. Of these, six were correlated with tumor shrinkage and PFS. Abbreviations: miRNA = microRNA, FDR = false discovery rate, HIF-2α = hypoxia-induced factor 2α, VEGFR1 = vascular endothelial growth factor receptor 1; VEGFR2 = vascular endothelial growth factor receptor 2, PFS = progression-free survival, VEGFR-TKI = vascular endothelial growth factor receptor tyrosine kinase inhibitor, miR = microRNA.

Sequencing data of 454 miRNAs were available in all tumor samples. As the biological function of miRNA is to inhibit mRNA levels or translation, only miRNAs correlated with lower mRNA expression were considered for further study resulting in 94 miRNA–mRNA correlations. Full correlation results are reported in Table S2. These miRNA–mRNA interactions were then searched in the miRDB, miRabel and DIANA Tarbase v.8 miRNA–mRNA interaction databases (Table 2 and Table S3) [22,23,24]. miRNAs with predicted target sequences in HIF-2α, VEGFR1 and/or VEGFR2 are visualized in Figure S1. For five of the miRNAs correlated with HIF-2α, the interaction with HIF-2α was listed by miRNA–mRNA interaction databases. For miR-142-5p, miR-185-5p and miR-223-3p, this interaction was also experimentally validated [26,27,28,29,30]. We identified nine miRNAs correlated with VEGFR1 for which this potential interaction was also listed by miRNA–mRNA databases. Of these, miR-142-3p and miR-378a-3p had experimental validation of the interaction with VEGFR1 [31,32,33]. For VEGFR2, we identified five miRNAs correlated with lower expression that were also mentioned as targeting VEGFR2 in the aforementioned databases. Studies providing experimental validation were found for miR-21-3p, miR-221-3p and miR-370-3p [15,34,35,36,37]. In summary, we selected 14 miRNAs with 19 miRNA–mRNA interactions (Figure 1).

### 3.3. Correlation of miRNAs with Tumor Shrinkage, PFS and OS

**Figure 2:**
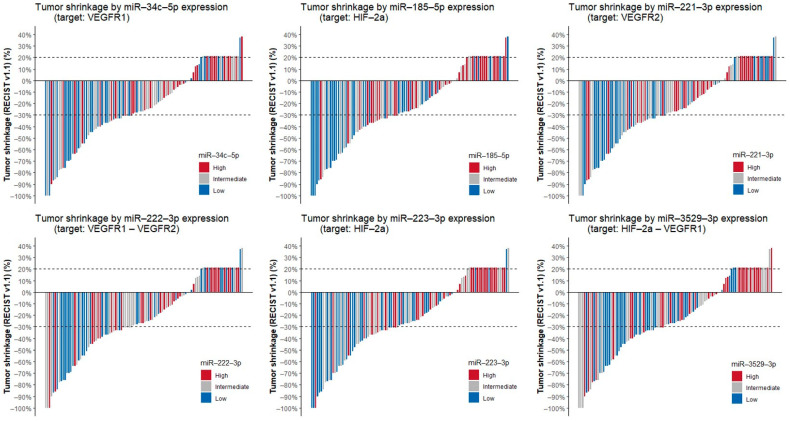
Waterfall plots displaying tumor shrinkage by miRNA expression per sample as evaluated by RECIST v1.1 criteria. For visual representation, miRNA expression levels were divided into high, intermediate and low expression by terciles of the respective miRNA expression levels. Abbreviations: miR = microRNA, VEGFR1 = vascular endothelial growth factor receptor 1, RECIST v.1.1 = Response Evaluation Criteria In Solid Tumors, version 1.1, HIF-2α = hypoxia-induced factor 2α, VEGFR2 = vascular endothelial growth factor receptor 2.

We evaluated the impact of the expression of these 14 miRNAs on clinical outcomes of patients treated with first-line VEGFR-TKIs, as displayed in Table 3. We show that nine miRNAs are associated with shorter PFS with first-line VEGFR-TKIs. A multivariable Cox proportional hazards model including miRNA expression and IMDC risk category as covariates showed that for miR-185-5p, miR-221-3p and miR-3529-3p, the effect on PFS is independent of IMDC risk category (Table S4). A total of nine miRNAs are associated with worse OS since start of first-line therapy. For miR-21-3p, miR-34c-5p, miR-149-5p, miR-185-5p and miR-3529-3p this association was independent of IMDC category on multivariable Cox proportional hazards analysis (Table S5). Of the 14 miRNAs, six were correlated with tumor shrinkage by Spearman correlation, indicating that for these miRNAs, higher miRNA expression was associated with less tumor shrinkage under treatment with VEGFR-TKIs. Waterfall plots displaying tumor shrinkage for these six miRNAs are shown in Figure 2. Correlation plots of the expression levels of these miRNAs with their respective targets are displayed in Figure S2. As these six miRNAs are correlated with both PFS and tumor shrinkage, they might be predictive of VEGFR-TKIs efficacy.

### 3.4. miRNA Expression in Molecular Subclassification ccrcc1-4

**Figure 3:**
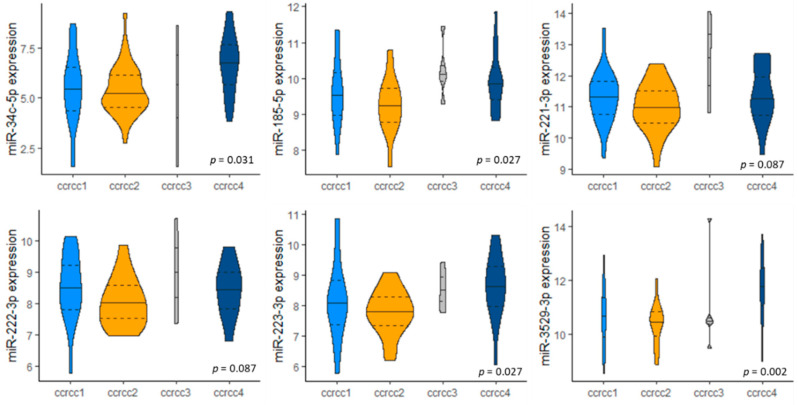
Violin plots of miRNA expression by molecular subtype ccrcc1-4. Note: Molecular subtypes, based on transcriptome data were available for 76 samples. Mean values are indicated by a full horizontal line; quartiles are indicated by dashed horizontal lines. *p*-values as calculated by Kruskal–Wallis test and FDR corrected. Abbreviations: miR = microRNA.

Finally, we investigated whether the expression of the six selected miRNAs differed between the groups of our previously reported molecular subclassification ccrcc1-4 [18] (Figure 3). miR-34c-5p, miR-185-5p, miR-223-3p and miR-3529-3p are expressed differently between subtypes; for miR-221-3p and miR-222-3p this trend did not reach statistical significance (Kruskal–Wallis test; *p*-values are FDR corrected). All six miRNAs had lower expression in the ccrcc2 subtype when compared to other subtypes (ccrcc1, ccrcc3 and ccrcc4) (Mann–Whitney U test; *p*-values are FDR corrected) (Table S6).

## 4. Discussion

Increased angiogenesis is one of the metabolic hallmarks of m-ccRCC. VEGFR-TKIs are one of the cornerstones of m-ccRCC treatment, both in combination with immune checkpoint inhibitors in first-line therapy or as single agents in further treatment lines. The HIF-2α inhibitor belzutifan is currently investigated in clinical trials and is showing promising efficacy [5,6,7]. However, clinically useful biomarkers to predict prognosis or response on anti-angiogenic therapies are currently lacking [38].

**Figure 4:**
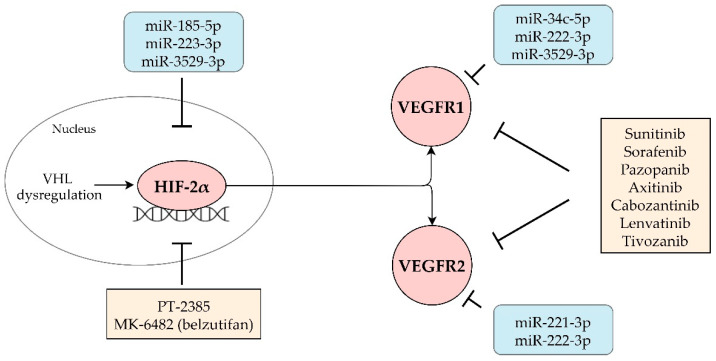
Through targeting HIF-2α, VEGFR1 and/or VEGFR2, miRNAs can deplete the drug targets of HIF-2α inhibitors and/or VEGFR-TKIs and lead to therapeutic resistance. Abbreviations: HIF-2α = hypoxia-induced factor- 2α, VEGFR1 = vascular endothelial growth factor receptor 1, VEGFR2 = vascular endothelial growth factor receptor 2, miRNA = microRNA, VEGFR-TKIs = vascular endothelial growth factor receptor tyrosine kinase inhibitors, miR = microRNA.

In this study, we identified four miRNAs (miR-34c-5p, miR-221-3p, miR-222-3p and miR-3529-3p) associated with reduced VEGFR1 and/or VEGFR2 expression and with tumor shrinkage and PFS upon treatment with VEGFR-TKIs, indicative of the potential predictive value of these miRNAs. We also identified three miRNAs (miR-185-5p, miR-223-3p, miR-3529-3p) associated with reduced HIF-2α expression and with tumor shrinkage and PFS with VEGFR-TKIs. These three miRNAs can be predictive not only for response to VEGFR-TKIs but potentially also for treatment with the HIF-2α inhibitor belzutifan (Figure 4). These six miRNAs had lower expression levels in the ccrcc2 molecular subtype, which is characterized by increased angiogenesis, a more indolent tumor behavior and higher sensitivity to VEGFR-TKIs [17].

Through targeting HIF-2α, VEGFR1 and/or VEGFR2, these six miRNAs could deplete the drug targets of HIF-2α inhibitors and/or VEGFR-TKIs, explaining inferior outcomes of anti-angiogenic therapies in m-ccRCC. However, in cell lines of other malignancies without the context of VEGFR-TKIs, overexpression of these miRNAs might reduce tumor growth through their anti-angiogenic effects; thus, other literature findings of correlations with outcomes are to be interpreted with caution.

### 4.1. miR-34c-5p Targets VEGFR1

In our study, higher expression of miR-34c-5p is correlated with reduced levels of VEGFR1 mRNA, less tumor shrinkage and worse PFS with first-line TKIs and worse OS. Few studies have investigated its effect on angiogenesis. In bladder cancer, miR-34c-5p enhances proliferation and migration of malignant cells through NOTCH1 targeting [39]. miR-34c-5p also enhances oncogenesis in colorectal cancer [40]. In other malignancies such as non-small cell lung cancer, laryngeal squamous cell cancer and cervical cancer, a tumor suppressive role is observed [41,42,43]. A miR-34a mimic demonstrated proof-of-concept in a phase I clinical trial for advanced solid tumors, including RCC, through dose-dependent modulation of target genes. Although initial results were promising, severe immune-mediated adverse events resulted in early closure of the trial [44].

### 4.2. miR-185-5p Targets HIF-2α

HIF-2α is a predicted and experimentally validated target of miR-185-5p, which is consistent with our findings that miR-185-5p is inversely correlated with HIF-2α expression [28,29]. In ccRCC, miR-185-5p expression levels are higher in tumor tissues when compared to normal kidney samples [45]. The anti-angiogenic effect of miR-185-5p has been demonstrated in prostate cancer cells, in which it led to suppression of tumor development [46]. Its inhibition of angiogenesis has also been described in benign diseases such as polycystic ovarian syndrome through targeting of VEGFA [47]. In endometriosis, a disease characterized by increased angiogenesis, miR-185-5p is downregulated in plasma samples [48]. In the current study, we evaluated the impact of miRNA expression on outcomes of VEGFR-TKIs. Here, miR-185-5p negatively impacted PFS, tumor shrinkage and OS, which is consistent with the hypothesis that it downregulates tumoral HIF-2α. However, when its effect is studied in a treatment-naïve context, a tumor-suppressor effect could be seen as angiogenesis inhibition is detrimental to tumor growth. This could explain why other studies have observed shrinkage and apoptosis of ccRCC tumor cells in vitro when miR-185-5p is overexpressed [49]. In hepatocellular carcinoma [50], prostate cancer [51] and colon cancer [29], miR-185-5p leads to increased apoptosis and inhibits cell invasion. However, some conflicting evidence exists as a previous study has demonstrated a positive correlation between miR-185-5p, high microvascular density and VEGFR2 mRNA expression levels in 82 ccRCC samples [52].

### 4.3. miR-221-3p and miR-222-3p Both Target VEGFR2, miR-222-3p Targets VEGFR1

miR-221-3p and miR-222-3p originate from the same cluster, and they are highly homologous. VEGFR2 is a predicted and experimentally validated target of miR-221-3p and miR-222-3p [15,37]. Additionally, VEGFR1 is also listed as a potential target of miR-222-3p. In our findings, miR-221-3p and miR-222-3p were correlated with lower expression of VEGFR2 and associated with worse PFS and tumor shrinkage. miR-221-3p expression was also correlated with worse OS. Both have lower expression in the angiogenic ccrcc2 molecular subtype. Several previous studies demonstrated the negative impact of miR-221/222 on VEGFR-TKI efficacy. A study of 30 RCC patients treated with sunitinib showed poorer PFS with increased miR-221/222 expression [15,53]. Overexpression of miR-221/miR-222 was also associated with poorer PFS and progressive disease upon treatment with VEGFR-TKIs in a study of 74 ccRCC patients [19]. In prostate adenocarcinoma, miR-221-3p has already been proposed as a possible escape mechanism from VEGFR2 inhibition [37]. Extensive evidence supports the anti-angiogenic role of the miR-221/222 cluster in endothelial cells [54,55,56,57]. Further studies on the oncogenic properties of the miR-221/222 cluster showed that miR-221-3p is upregulated in ccRCC tissues [45]. It has higher plasma concentrations in RCC patients when compared to controls [58]. Two studies have demonstrated worse outcomes in ccRCC patients with higher levels of circulating miR-221, highlighting its potential for liquid biopsies [59]. miR-222 also has higher serum levels in breast cancer patients [60] and has been proposed as a plasma biomarker for lymph node metastasis in thyroid carcinoma [61]. Moreover, the miR-221/222 cluster enhances tumorigenesis in breast cancer [62]. In hepatocellular carcinoma, miR-221 overexpression was associated with more advanced tumor stage and worse outcome [63]. In endometrial carcinoma, lung cancer and liver cancers, miR-222 promotes proliferation and invasion [64,65]. A meta-analysis on miR-221 and miR-222 pooling results from 6086 patients across different cancer types concluded a worse prognosis for patients with higher expression of miR-222, in terms of both OS and secondary outcomes such as disease-free survival and recurrence-free survival [66]. Some contradictory evidence exists, as a previous study of 56 m-ccRCC patients treated with sunitinib showed that tumoral miR-221 expression showed no association with sunitinib response [67]. In a study of 74 ccRCC patients with tumor thrombosis, miR-221 was not significantly associated with cancer-specific survival [68]. Moreover, silencing of the miR-221/222 cluster decreases angiogenesis in glioblastoma [69].

### 4.4. miR-223-3p Targets HIF-2α

In our study, miR-223-3p is correlated with lower expression of HIF-2α, which is consistent with previous studies in lung ischemia/reperfusion injuries demonstrating that miR-223 directly targets HIF-2α [30]. Additionally, miR-223-3p is associated with shorter PFS and OS and less tumor shrinkage with VEGFR-TKIs. The anti-angiogenic effects of miR-223-3p were previously described in head and neck squamous cell cancer [70]. In ischemic cardiac microvascular endothelial cells, miR-223-3p inhibits angiogenesis through the RPS6KB1/HIF-1α signal pathway [71]. Overexpression of miR-223-3p has previously been associated with higher tumor grades and stages and worse clinical outcomes in ccRCC patients, additionally it was also associated with increased cell proliferation and metastasis in ccRCC cell lines [72]. Two other studies demonstrated higher expression of miR-223-3p in ccRCC tissues when compared to paracancerous tissues. Moreover, tumoral overexpression was associated with worse OS [11,13]. In lung cancer cells, it promotes tumor invasiveness through targeting EPB41L3 [73]. In breast cancer, a more tumor suppressive role is suggested [74], even though it is upregulated in serum samples from breast cancer patients when compared to healthy controls [60,75].

### 4.5. miR-3529-3p Targets Both HIF-2α and VEGFR1

We demonstrated an inverse correlation of miR-3529-3p with expression of both HIF-2α and VEGFR1, which are predicted targets of miR-3529-3p. Moreover, it is also correlated with worse PFS and less tumor shrinkage with VEGFR-TKIs. miR-3529-3p overexpression also negatively impacts OS. miR-3529-3p is upregulated in radiotherapy-resistant colorectal cancer cells [76]. To our best knowledge, this is the first study to demonstrate an association between miR-3529-3p and angiogenesis.

## 5. Conclusions

We identified four miRNAs (miR-34c-5p, miR-221-3p, miR-222-3p and miR-3529-3p) inversely correlated with VEGFR1 and/or VEGFR2 expression and associated with less tumor shrinkage and shorter PFS upon treatment with VEGFR-TKIs. Moreover, we identified three miRNAs (miR-185-5p, miR-223-3p and miR-3529-3p) inversely correlated with HIF-2α expression and associated with less tumor shrinkage and shorter PFS upon treatment with VEGFR-TKIs. These miRNAs could serve as biomarkers for established VEGFR-TKI treatments or upcoming HIF-2α inhibitors. Further prospective studies investigating the predictive potential of these miRNAs are warranted.

## Figures and Tables

**Table 1:** Patient characteristics.

**All Patients**	***n* = 109**	
Gender: male	70	64.2%
Gender: female	39	35.8%
Median age at diagnosis (years)	62	IQR: 55–68
Median OS after diagnosis (months)	46	IQR: 21–110
Median OS after stage IV (months)	32	IQR: 15–64
**IMDC risk group at start of first-line therapy**		
Favorable (*n*)	13	11.9%
Intermediate (*n*)	69	63.3%
Poor (*n*)	27	24.8%
**First-line targeted therapy**		
Sunitinib (*n*)	68	62.4%
Pazopanib (*n*)	30	10.1%
Sorafenib (*n*)	11	27.5%
**Molecular subtypes**		
ccrcc1	19/76	25%
ccrcc2	33/76	43.4%
ccrcc3	4/76	5.2%
ccrcc4	20/76	26.3%

Abbreviations: IQR = interquartile range, OS = overall survival, IMDC = International Metastatic RCC Database Consortium. Note: Molecular subtype proportions are relative to the total number of patients for whom mRNA expression data were available (*n* = 76).

**Table 2:** miRNAs inversely correlated with HIF-2α, VEGFR1 or VEGFR2 expression and listed by miRNA–mRNA interaction databases.

miRNA	Interaction with HIF-2α				Interaction with VEGFR1				Interaction with VEGFR2			
	*rho*	*p*-Value	Interaction Database	Experimental Validation	*rho*	*p*-Value	Interaction Database	Experimental Validation	*rho*	*p*-Value	Interaction Database	Experimental Validation
miR-21-3p	**0.375**	**0.006**	**yes ^3^**	–	0.355	0.009	–	–	**0.317**	**0.022**	**yes ^3^**	[34]
miR-142-5p	**0.305**	**0.028**	**yes ^1,2,3^**	[26,27]	0.199	0.182	–	–	0.247	0.085	–	–
miR-185-5p	**0.324**	**0.019**	**yes ^2,3^**	[28,29]	0.405	0.003	–	–	0.342	0.012	–	–
miR-223-3p	**0.300**	**0.031**	**yes ^2^**	[30]	0.357	0.008	–	–	0.335	0.014	–	–
miR-3529-3p	**0.330**	**0.016**	**yes ^1^**	–	**0.358**	**0.008**	**yes ^1^**	–	0.297	0.032	–	–
miR-34c-5p	0.293	0.035	–	–	**0.366**	**0.006**	**yes ^3^**	–	0.301	0.031	–	–
miR-142-3p	0.350	0.010	–	–	**0.285**	**0.041**	**yes ^2,3^**	[31]	0.292	0.035	–	–
miR-149-5p	0.273	0.052	–	–	**0.296**	**0.032**	**yes ^1,2^**	–	**0.316**	**0.022**	**yes ^1,2^**	–
miR-222-3p	0.355	0.008	–	–	**0.417**	**0.002**	**yes ^3^**	–	**0.410**	**0.002**	**yes ^1,2^**	[15]
miR-370-3p	0.254	0.075	–	–	**0.347**	**0.010**	**yes ^1^**	–	**0.280**	**0.047**	**yes ^1,2^**	[35,36]
miR-378a-3p	0.270	0.054	–	–	**0.284**	**0.043**	**yes ^1^**	[32,33]	0.235	0.104	–	–
miR-664a-5p	0.270	0.055	–	–	**0.282**	**0.044**	**yes ^1^**	–	0.241	0.094	–	–
miR-1301-3p	0.230	0.114	–	–	**0.340**	**0.013**	**yes ^2^**	–	0.271	0.054	–	–
miR-221-3p	0.240	0.095	–	–	0.337	0.014	–	–	**0.332**	**0.016**	**yes ^1,2^**	[37]

Abbreviations: miRNA = microRNA, HIF-2α = hypoxia-induced factor 2α, VEGFR1 = vascular endothelial growth factor receptor 1; VEGFR2 = vascular endothelial growth factor receptor 2, miR = microRNA. Note: Correlation coefficient rho as calculated by Spearman correlation. As mRNA expression is reported in Ct values, for which higher values indicate lower mRNA expression, positive correlation coefficients indicate that miRNA expression is correlated with lower mRNA expression. Reported *p*-values are FDR-corrected. Predicted correlations are highlighted in bold. ^1^: Interaction listed by miRDB [22]; ^2^: interaction listed by miRabel database with RRA score < 0.05 [23]; ^3^: interaction listed by DIANA Tarbase v8 [24].

**Table 3:** Correlation of 14 miRNAs with tumor shrinkage, PFS and OS since start of first-line VEGFR-TKI therapy.

miRNA	Tumor Shrinkage		PFS		OS	
	*rho*	*p*-Value	HR (95% CI)	*p*-Value	HR (95% CI)	*p*-Value
miR-21-3p	0.127	0.260	**1.25 (1.05–1.49)**	**0.025**	**1.37 (1.14–1.63)**	**0.002**
**miR-34c-5p**	**0.356**	**0.003**	**1.20 (1.06–1.36)**	**0.013**	**1.26 (1.11–1.44)**	**0.002**
miR-142-3p	0.153	0.198	1.08 (0.91–1.27)	0.431	1.12 (0.95–1.32)	0.197
miR-142-5p	0.147	0.198	1.03 (0.85–1.24)	0.798	1.03 (0.85–1.25)	0.770
miR-149-5p	0.151	0.198	**1.44 (1.13–1.83)**	**0.012**	**1.57 (1.23–2.00)**	**0.002**
**miR-185-5p**	**0.277**	**0.022**	**1.70 (1.33–2.17)**	**<0.001**	**1.77 (1.37–2.27)**	**<0.001**
**miR-221-3p**	**0.254**	**0.028**	**1.58 (1.21–2.05)**	**0.006**	**1.36 (1.07–1.74)**	**0.025**
**miR-222-3p**	**0.233**	**0.043**	**1.31 (1.04–1.66)**	**0.035**	1.28 (1.03–1.60)	0.053
**miR-223-3p**	**0.334**	**0.004**	**1.28 (1.04–1.58)**	**0.035**	**1.33 (1.07–1.65)**	**0.025**
miR-370-3p	−0.005	0.963	1.14 (1.01–1.30)	0.055	**1.17 (1.03–1.33)**	**0.038**
miR-378a-3p	0.034	0.789	1.15 (0.92–1.44)	0.293	1.22 (0.98–1.53)	0.106
miR-664a-5p	0.107	0.330	1.14 (0.89–1.47)	0.357	1.19 (0.93–1.53)	0.183
miR-1301-3p	0.181	0.138	**1.42 (1.08–1.86)**	**0.025**	**1.45 (1.10–1.91)**	**0.025**
**miR-3529-3p**	**0.255**	**0.028**	**1.38 (1.13–1.69)**	**0.007**	**1.44 (1.18–1.76)**	**0.002**

Abbreviations: miRNA = microRNA, PFS = progression-free survival, OS = overall survival, VEGFR-TKI = vascular endothelial growth factor receptor tyrosine kinase inhibitor, miR = microRNA, HR = hazard ratio, CI = confidence interval. Note: Correlation coefficient with tumor shrinkage as calculated with Spearman correlation test. HR and CI as calculated with Cox proportional hazard model. Reported *p*-values are FDR-corrected. Significant results are highlighted in bold. miRNA correlated with both tumor shrinkage and PFS are highlighted in bold.

## Data Availability

The miRNA-seq data have been deposited in the ArrayExpress database at EMBL-EBI (www.ebi.ac.uk/arrayexpress) under accession number E-MTAB-10586. The mRNA qRT-PCR data have also been deposited in the ArrayExpress database under the accession number E-MTAB-10592.

## Supplementary Materials

The following are available online at https://www.mdpi.com/article/10.3390/cancers13123099/s1, Figure S1: Network visualization of miRNA–mRNA target interactions as listed by miRDB, miRabel (RRA < 0.05) and Diana Tarbase v.8, Figure S2: Correlation of miRNA with mRNA expression, Table S1: Included patients with clinical data and sample IDs used in ArrayExpress submissions, Table S2: Correlation of miRNA with mRNA expression (Spearman correlation), Table S3: miRNA–mRNA target interactions as listed by miRDB, miRabel (RRA < 0.05) and Diana Tarbase v.8, Table S4: Correlation of miRNA expression with progression-free survival (multivariable Cox proportional hazards model with miRNA expression and IMDC category), Table S5: Correlation of miRNA expression with overall survival since start of first-line therapy (multivariable Cox proportional hazards model with miRNA expression and IMDC category), Table S6: miRNA expression by molecular subtype ccrcc1-4.
