# Supplementary figures and images for: MicroRNAs Targeting HIF-2α, VEGFR1 and/or VEGFR2 as Potential Predictive Biomarkers for VEGFR Tyrosine Kinase and HIF-2α Inhibitors in Metastatic Clear-Cell Renal Cell Carcinoma
Source: Cancers (Basel). 2021 Jun 21;13(12):3099. doi: 10.3390/cancers13123099 (PMC8235409; doi:10.3390/cancers13123099)

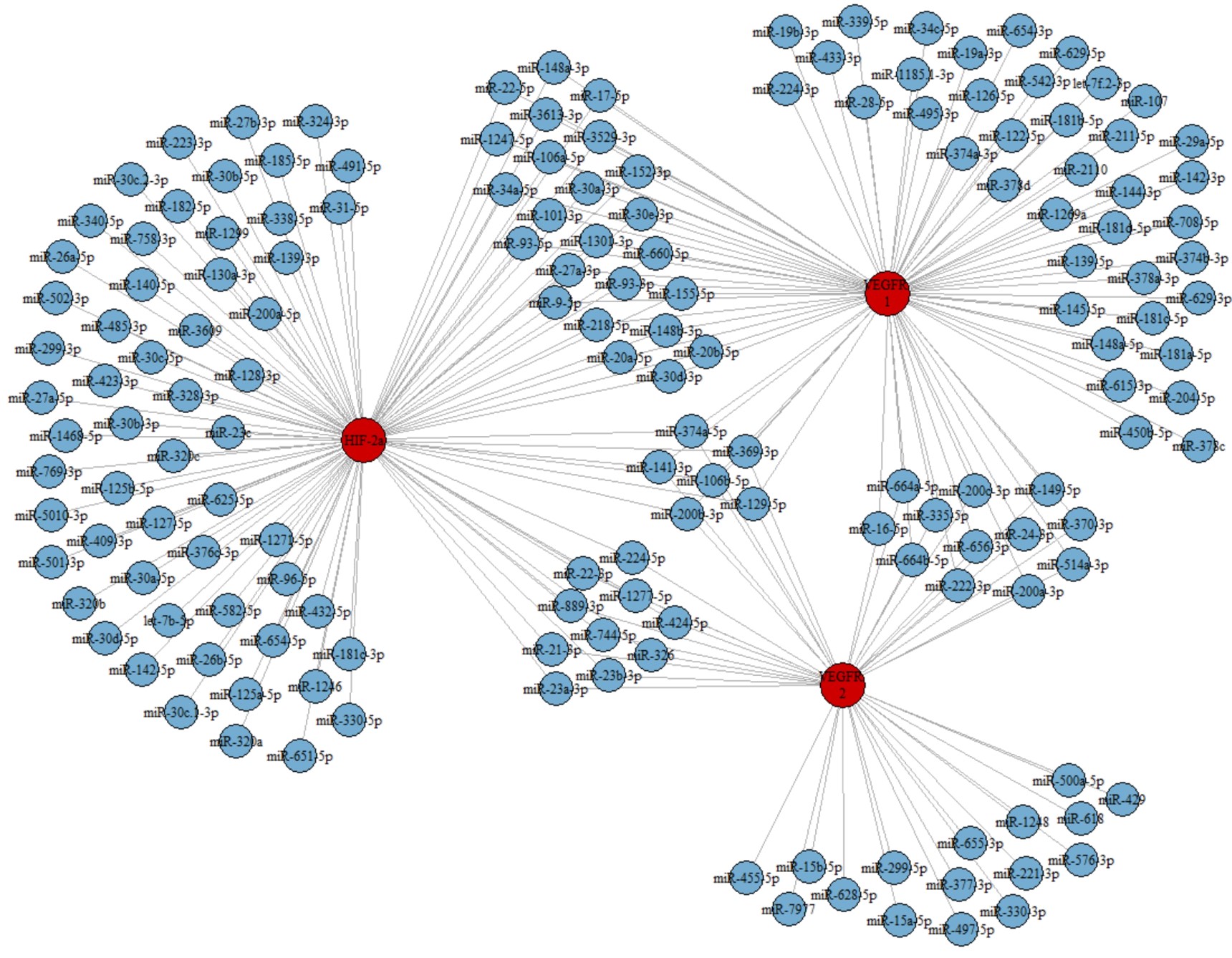

Supplement: Supplementary file 1 [file cancers-13-03099-s001.zip › Figure S1.jpg]

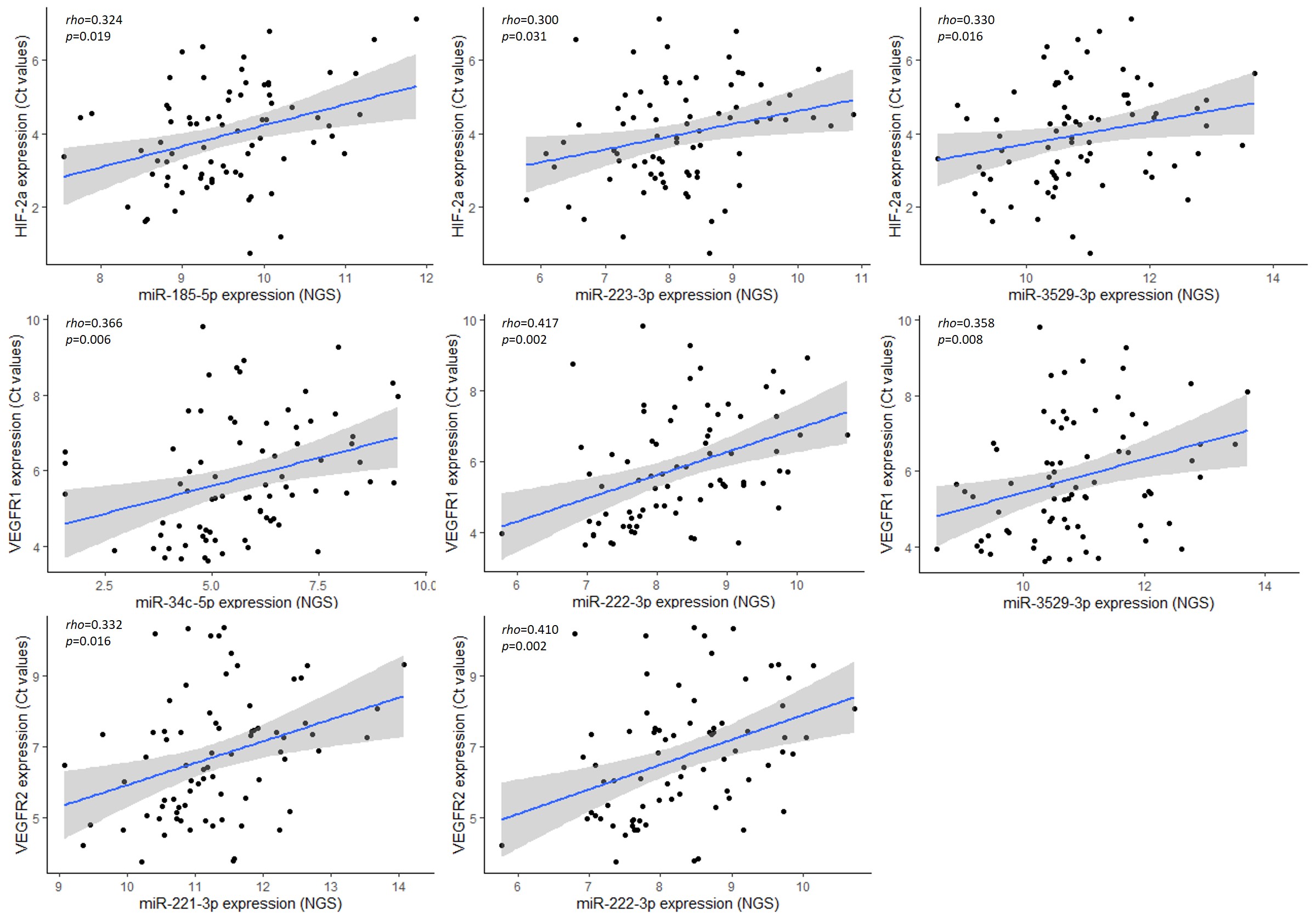

Supplement: Supplementary file 1 [file cancers-13-03099-s001.zip › Figure S2.jpg]
